# Clinical Outcome and Our Experience of 33 Consecutive Cases Treated With Cervical Microendoscopic Laminoplasty in a Single Clinic

**DOI:** 10.7759/cureus.83917

**Published:** 2025-05-11

**Authors:** Keita Kuraishi, Yoshinori Maki, Toshinari Kawasaki, Tamaki Kobayashi, Yoshihiko Ioroi

**Affiliations:** 1 Department of Spinal Surgery, Ohmi Sebone Clinic, Omihachiman, JPN; 2 Neurosurgery, Ayabe Renaiss Hospital, Ayabe, JPN; 3 Neurosurgery, Hikone Chuo Hospital, Hikone, JPN; 4 Neurosurgery, Kyoto Katsura Hospital, Kyoto, JPN; 5 Spinal Surgery, Kyoto Katsura Hospital, Kyoto, JPN; 6 Department of Neurosurgery, Kyoto Katsura Hospital, Kyoto, JPN

**Keywords:** less invasive surgery, microendoscopic laminoplasty, myelopathy, ossification of the posterior longitudinal ligament, radiculopathy

## Abstract

Introduction: Cervical microendoscopic laminoplasty (CMEL) is a less invasive operation for cervical myelopathy. This method is gradually spreading; however, the literature seems to have few reports concerning clinical outcomes. This study aims to report the effectiveness of CMEL performed in a private clinic.

Methods: Patients who underwent CMEL from April 2023 to July 2024 in our private clinic were retrospectively researched. The patient's background and the following operative data were collected, such as the number of vertebral levels treated with CMEL, additional foraminotomy, operative time, and intraoperative bleeding. Postoperative data were also collected, including admission days, complications, surgical outcomes, radiological changes, and follow-up periods. In this study, those patients with severe cardiovascular, pulmonary, and renal problems and/or significant motor weakness requiring postoperative rehabilitation were excluded.

Results: Consecutive 33 patients (30 spondylotic myelopathy and three ossification of the posterior longitudinal ligament) with a median age of 60 years were enrolled. CMEL was performed for a single level in 13 cases, two in 15, and three in five, respectively. Foraminotomy was concurrently performed in eight cases. The mean operative time and intraoperative bleeding were 105 minutes and 21 g, respectively. All the patients were discharged from the hospital the following day after surgery on their own without severe neck pain. Postoperative extradural hematoma occurred only in a single case, which was managed with an additional operation. The median postoperative follow-up period (minimum-maximum) was 9.5 (two to 18) months. The outcome evaluated with the McNab score was excellent (six cases), good (22 cases), fair (four cases), and poor (one case). The median C2-7 angle improved from 6.2° preoperatively to 8.1° postoperatively with no significant difference.

Conclusion: CMEL can be performed in a private clinic for patients without perioperative high risks.

## Introduction

Cervical posterior decompression surgery (i.e., laminectomy and laminoplasty) is a well-established method for cervical spondylosis, developmental spinal canal stenosis, and ossification of the posterior longitudinal ligament (OPLL) [1-3]. Typically, cervical laminoplasty was performed with a long skin incision, resulting in damage to the nuchal ligament and paravertebral muscles [1-3]. Axial neck pain and stiffness, kyphosis, and reduced range of motion are major concerns following conventional cervical laminoplasty [4-7]. Cervical laminoplasty with a small skin incision and less damage to the nuchal ligament and paravertebral muscles was described; however, postoperative neck discomfort remains [8].

As the microendoscope has also been applied in the cervical spinal field since 2000 [9], cervical laminoplasty can be performed under the guidance of a microendoscope [10]. Minamide et al. described the efficacy of cervical microendoscopic laminoplasty (CMEL) for cervical myelopathy [10]. This less-invasive surgical approach is considered equivalent to conventional cervical laminectomy, based on the results of postoperative follow-up two and five years after surgery [11,12]. Alternative CMEL is also proposed by Zhang et al. [13]. However, to the best of our knowledge, the description of CMEL performed in private clinics, not hospital settings, seems scant to date. The clinical data of CMEL practiced in private clinics can also contribute to the feasibility of implementation in the clinical field and the potential for widespread adoption. This can also lead to patients' benefits related to the less invasive nature of CMEL.

In this study, we report the postoperative outcomes of CMEL performed in our private clinic, referring to the previous literature.

## Materials and methods

**Figure 1:**
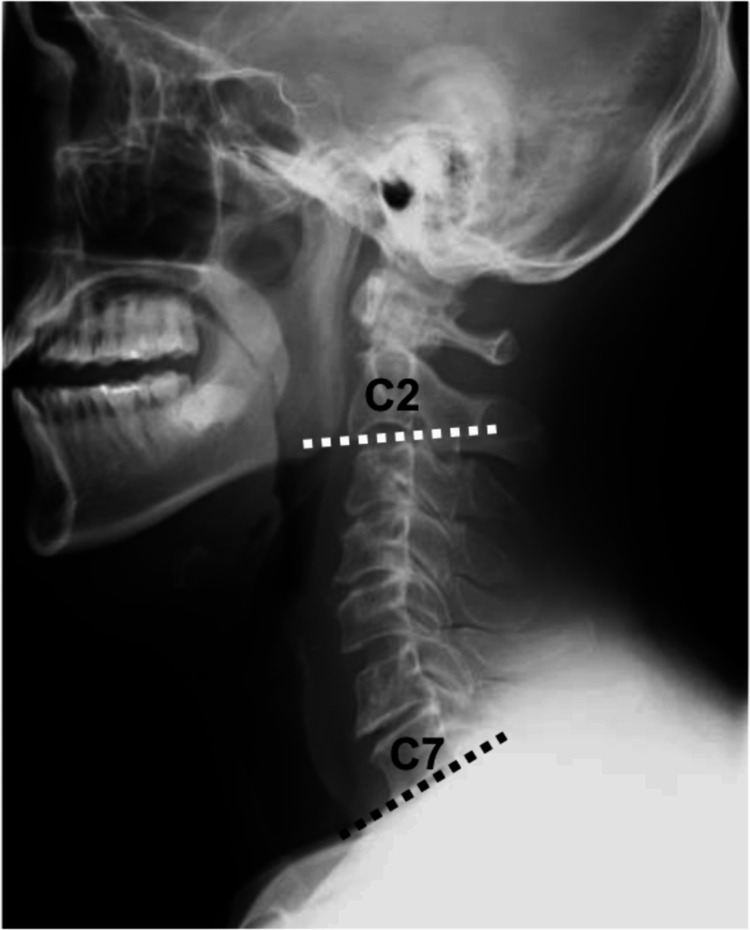
The definition of the C2-C7 angle. The angle between the lower endplate of C2 (white dotted line) and that of C7 (black dotted line) was measured pre- and postoperatively.

We enrolled daily independent patients with cervical myelopathy who underwent CMEL in Ohmi Sebone Clinic from April 2023 to July 2024. We searched the following variables from medical records: age, sex, number of vertebral levels treated with CMEL in an individual case, additional foraminotomy, operative time, intraoperative bleeding, admission days, postoperative complications, and follow-up periods. Pre- and postoperative radiological findings were evaluated with a C2-C7 angle (Figure 1).

The surgical outcomes were evaluated using the McNab score [14]. Patients included in this study were independent in their daily living. Patients with severe cardio, pulmonary, and renal problems were excluded, as perioperative management was difficult in our private clinic. Those patients with significant motor weakness in the lower extremities equivalent to manual muscle testing of 4 or less were excluded, either because rehabilitation therapy for admitted patients is not available in our clinic. This study was approved by the Ethical Committee of Ohmi Sebone Clinic (approval number: 2025-01, and approved on January 15, 2025).

CMEL procedure with/without foraminotomy

**Figure 2:**
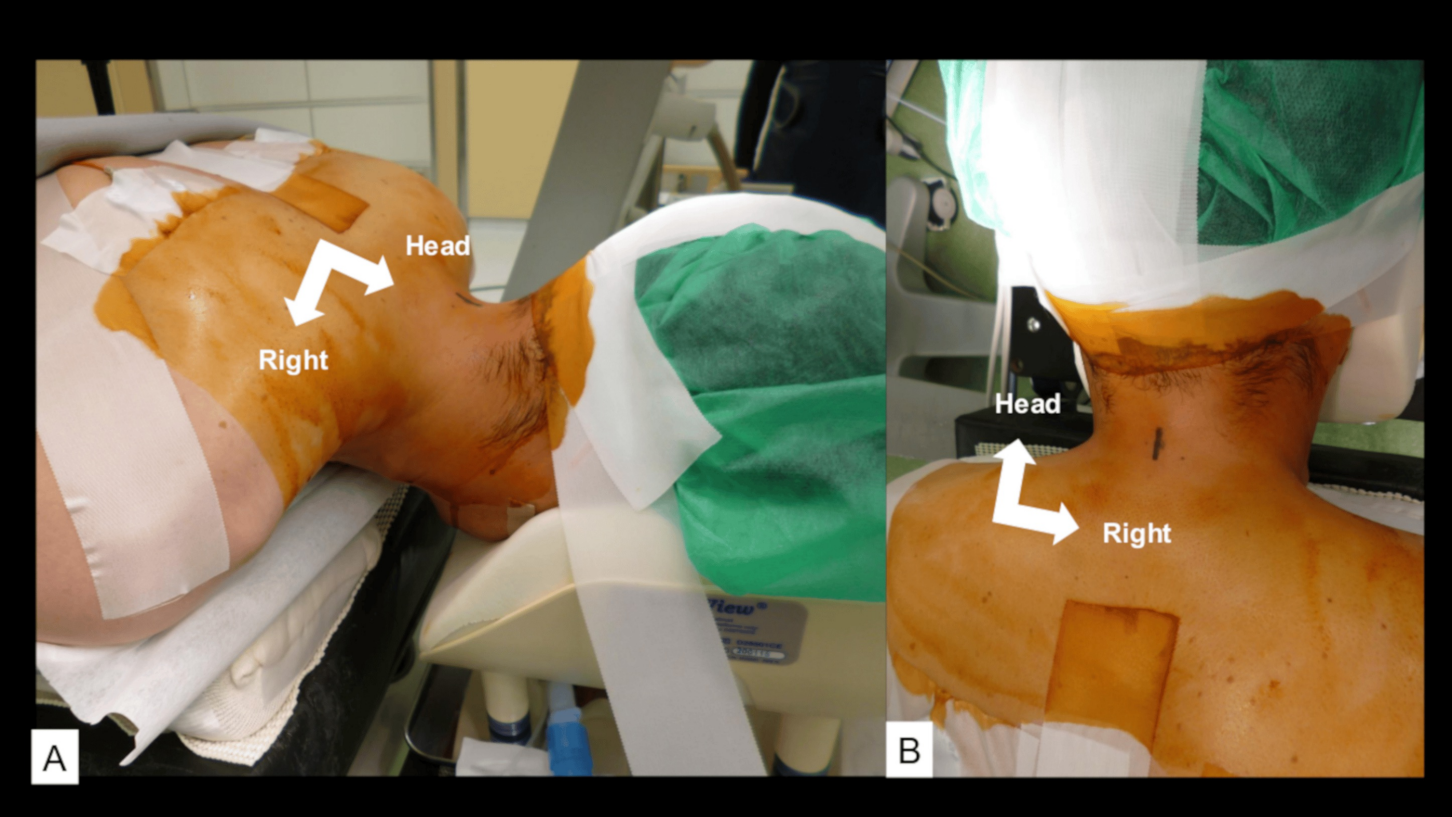
Intraoperative posture of the patient and design of the skin incision. The patient’s head is slightly raised, and the neck is set in the neutral position. The head is taped to the cushion without being pinned. A midline 18 mm skin incision is made.

This procedure was performed entirely by endoscopy. The patient was set in a prone position under general anesthesia. The head was slightly elevated, and the neck was neutral (Figure 2).

We confirmed the target vertebral levels under a fluoroscopy. A midline skin incision of approximately 18mm was made. The nuchal ligament was preserved, and a longitudinal incision was made on the left side to reach the vertebral arch using a dilator. The paravertebral muscle on the target vertebrae was detached with an electric knife from the left side. The laminae and the medial side of the facets were exposed. A short-type METRxTM (Medtronic), 49 mm in depth and 16 mm in diameter, was inserted to obtain the surgical field. The entering side of the lamina, base of the spinous process, and contralateral inner plate of the lamina were drilled using a 4 mm diamond drill, and hemilaminectomy was performed. In case the base of the spinous process was not large enough (usually at C3 and C4), the spinous process was completely removed or cut at the base to achieve decompression of the spinal cord.

The laminae of the most cranial and caudal-targeted vertebrae were also drilled along the margin of the yellow ligament. The yellow ligaments were removed, and the dura was exposed. In case there is a spinal cord compression lesion in two or more adjacent levels, the laminae were drilled from the cephalic laminae, and the caudal laminae were continuously drilled. The laminae were thinned to the utmost extent, then detached from the dura mater and the yellow ligaments. For the coexisted radiculopathy related to foraminal stenosis, we added foraminotomy. We usually entered from the left side of the patients, but entered from the right side only when right foraminal stenosis existed. After we achieved hemostasis using a low-power bipolar and microfibrillar collagen hemostat (Avitene, Becton, Dickinson and Company), a UK drain catheter of 10Fr (NIPRO) was inserted in the extradural space. The wound was closed.

## Results

A total of 33 consecutive patients (men:women = 17:16) were enrolled in this study. The median age (minimum-maximum) was 59.8 (42-83) years old. The median operated number of the vertebral levels was 1.76 (a single level in 13 cases, two in 15, and three in five, respectively). Skip laminectomy (i.e., C3/C4 and C5/C6) was performed in two cases, while laminectomy at multiple continuous levels was performed in 18 cases. Foraminotomy was concurrently performed in eight cases. Three cases of segmental-type OPLL were included. The mean operative time (minimum-maximum) was 105 (49-130) minutes. The mean intraoperative bleeding (minimum-maximum) was 21 (5-96) g. All the patients were discharged from the hospital the following day after surgery, and they did not complain of severe postoperative neck pain. Soft neck collars were prescribed for two patients as they wished. The median postoperative follow-up period (minimum-maximum) was 9.5 (two to 18) months. The outcome evaluated with the McNab score was excellent (six cases), good (22 cases), fair (four cases), and poor (one case: representative case 3). The four fair patients had concomitant diseases such as chronic inflammatory demyelinating polyneuropathy, fibromyalgia, and preoperative myelomalacia, manifesting pain in the upper extremities for over two years. C5 palsy was not observed in any case. The drainage tube was routinely removed the following day after surgery for the 23rd consecutive patient. However, after we experienced a case with postoperative extradural hematoma (representative case 3), we removed the drainage tube, confirming that the daily drained serum decreased below 30 ml. Then, the drainage tube was removed two or three days after surgery, and no hemorrhagic complication was observed. Postoperative progressive kyphosis was not observed. No additional operation, such as fixation surgery, was necessary except in the representative case 3. The median (minimum-maximum) pre- and postoperative C2-C7 angles were 6.2° (-18° - 30°), and 8.1° (-4° - 33°), respectively (Table 1).

**Table 1:** Results of 33 consecutive patients undergoing cervical microendoscopic laminoplasty *: p-value ＜0.05

Clinical variables	Results
Age (years old)	59.8 (42-83)
Sex (men/women) (cases)	17/16
Underlying disease (cases) Cervical spondylotic myelopathy/ossification of the posterior longitudinal ligament	30/3
Median surgical levels (a-single level decompression/two-level decompression/three-level decompression)	1.76 (13 cases/15 cases/5 case)
Foraminotomy (cases)	8
Operative time (minutes)	105 (49-134)
intraoperative bleeding (g)	21 (5-96)
Admission days (day)	1
MacNab scores (excellent/good/fair/poor) (cases)	6/22/4/1
Median pre/postoperative C2-7 angle (°)	6.2/ 8.1*
Follow-up period (months)	9.5 (2-18)

Representative cases

Case 1: A 48-Year-Old Man (C5/6 Myelopathy and Right C6 Radiculopathy)

**Figure 3:**
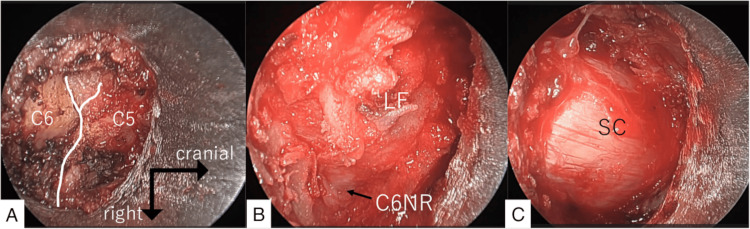
Intraoperative findings of Case1 treated with C5/6 cervical microendoscopic laminoplasty combined with microendoscopic foraminoplasty. A: The C5 and C6 laminae were exposed after detaching the muscle. B: After drilling the C5 and C6 laminae and the medial portion of the facet joint, the C6 nerve root was observed. C: Removing the ligamentum flavum, the spinal cord was completely decompressed. (LF: ligamentum flavum, NR: nerve root, SC: spinal cord)

He was complaining of pain in the bilateral upper extremities lasting for seven months. The pain radiated to the right thumb, corresponding to the dermatome of C6. The intervertebral disc at C5/6 compressed the cervical spinal cord and the right C6 nerve root. The right CMEL for C5 and C6 was simultaneously performed with cervical microendoscopic foraminoplasty (CMEF). The operation time was one hour and 40 minutes. Soon after surgery, the pain disappeared. The patient was discharged the following day. No symptoms recurred for 10 months (Figures 2, 3).

**Figure 4:**
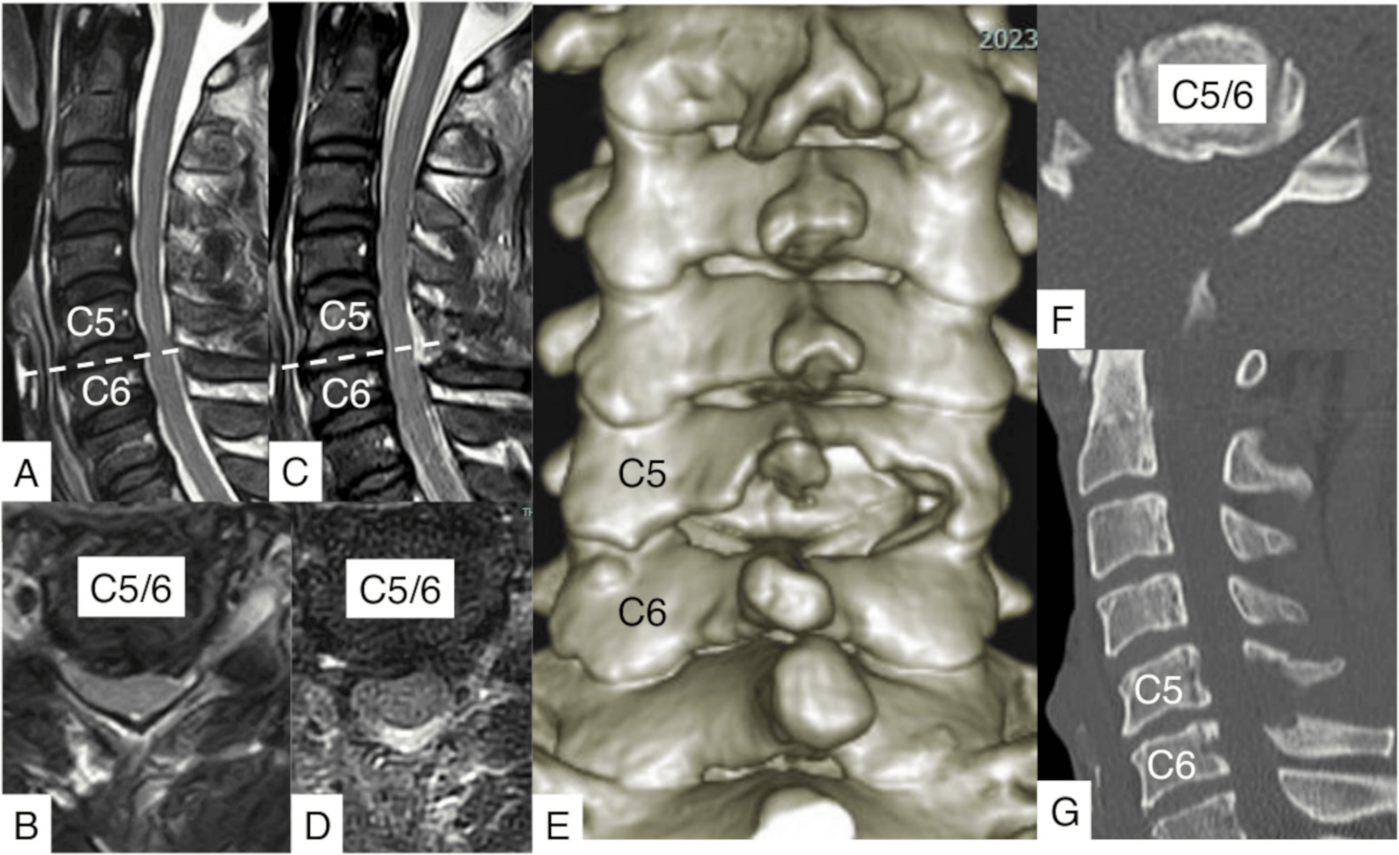
Pre- and postoperative radiological findings of Case 1. A, B: Preoperative magnetic resonance images (MRI) show C5/6 spinal cord compression and right foraminal stenosis because of disc herniation (A: sagittal image, B: axial image). C, D: Postoperatively, the spinal cord and right C6 nerve root were decompressed (C: sagittal image, D: axial image). E: Postoperative 3-dimensional reconstructed computed tomography (CT) and plain CT images show central decompression and foraminotomy at C5/C6. F, G: Postoperative CT images revealed posterior decompression at C5/C6 (F: axial image, G: sagittal image).

Case 2: A 60-Year-Old Woman (C4/5 and C6/7 Myelopathy, Skip Lesion)

The patient underwent anterior cervical discectomy and fusion at the C5/6 level 14 years before. Six years before, she started to feel numbness in the bilateral 1-3 fingers and pain in both shoulders with the extension position of the neck. Followingly, gait disturbance appeared. An MRI examination showed spinal cord compression at the levels of C4/5 and C6/7, which seemed to be adjacent-level complications after anterior fixation surgery. To resolve myelopathy at the C4/5 and C6/7 levels, skip CMEL was performed (Figure 4).

**Figure 5:**
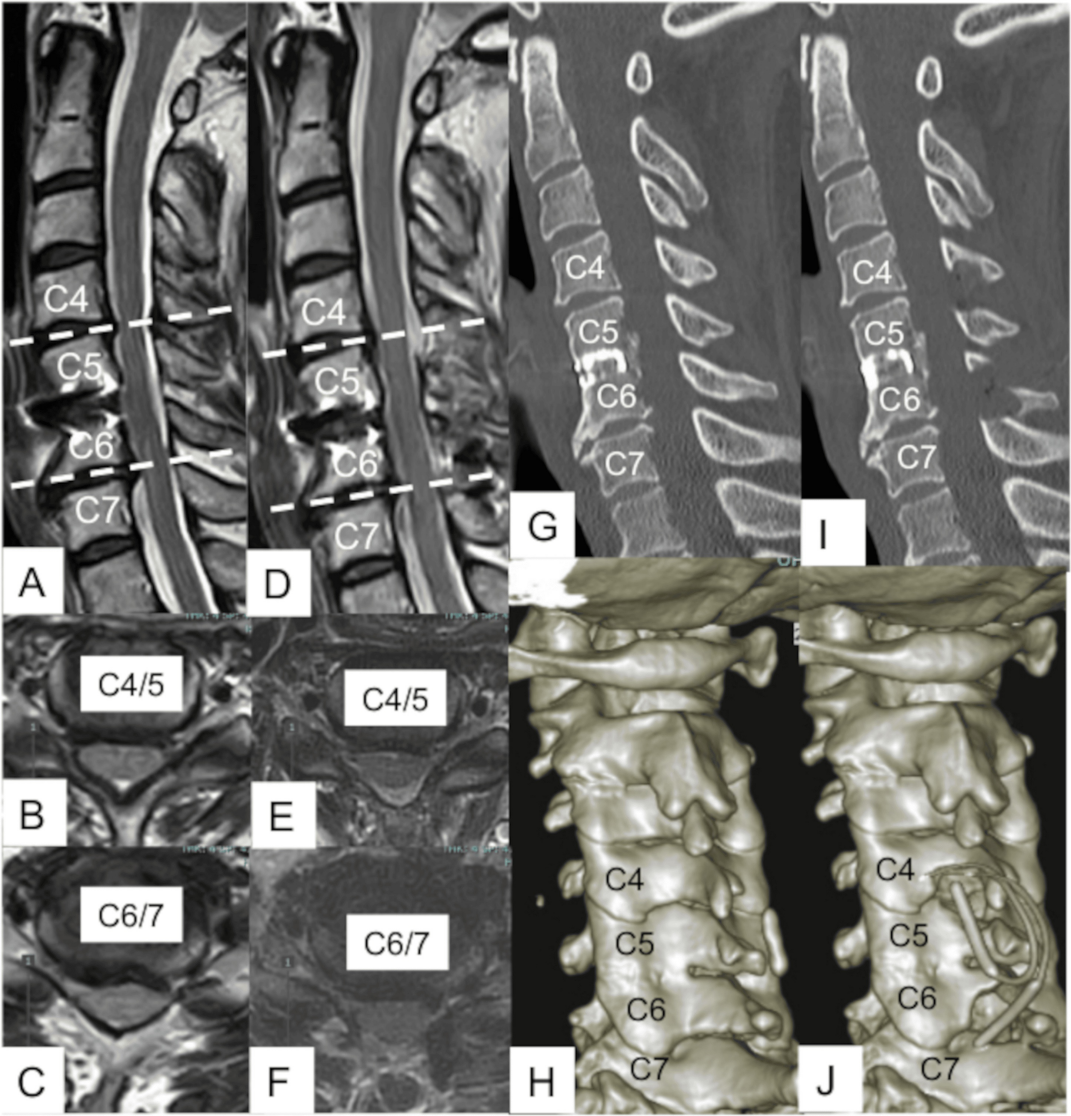
Pre- and postoperative radiological findings of Case 2. A-C: Preoperative magnetic resonance images (MRI) show C4/5 and C6/7 cord compression due to spinal canal stenosis and disc herniation (A: sagittal image, B, C: axial image). D-F: Postoperative MRIs show C4/5 and C6/7 decompression (D: sagittal image, E, F: axial image). G, H: Preoperative computed tomography images show C5/6 anterior fusion (G, a sagittal image, H: three-dimensional reconstructed image). I, J: Postoperative CT images show slip laminectomy at C4/5 and C6/7 (I, a sagittal image, J: three-dimensional reconstructed image).

After surgery, the numbness of the upper limbs improved, and the wobble of the lower limbs disappeared regardless of neck movement. No recurrence was observed in the postoperative 12 months.

Case 3: A 63-Year-Old Man (Postoperative Extradural Hematoma Case)

He suffered from ambulation difficulty and numbness in the upper and lower extremities. As severe cervical spinal stenosis from C5 to C7 was diagnosed, we performed CMEL for those levels. We also proposed the anterior approach to resolve cervical myelopathy; however, the patient refused this option because of the potential risks related to damage to the trachea, esophagus, or vessels. Postoperatively, the numbness in the upper extremities was ameliorated. The drainage tube was removed the following day, and the patient was discharged from the clinic, independently ambulating. However, two days later, he started complaining again of ambulation difficulty, numbness, and motor weakness in the upper extremities. An extradural hematoma was observed four days after surgery, hematoma removal was performed via the same skin incision using an endoscopy (Figure 5).

**Figure 6:**
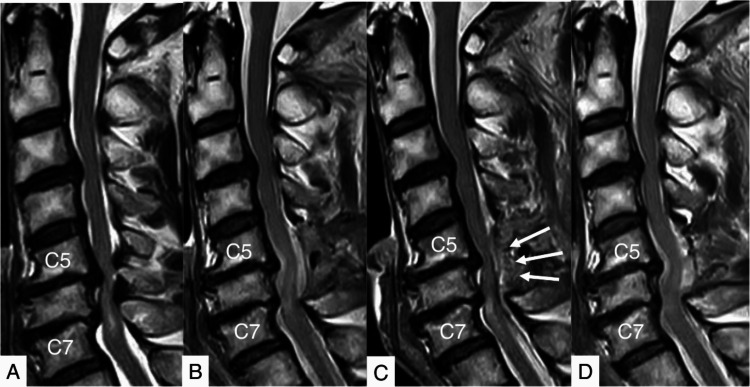
Pre- and postoperative magnetic resonance images (MRI) of Case 3. A: A preoperative sagittal MRI shows severe cord compression at C5/6 and C6/7. B: A postoperative sagittal MRI shows posterior decompression from C5 to C7. C: An extradural hematoma compressing the spinal cord (white arrows) was identified four days after surgery. D: The postoperative extradural hematoma was resolved after hematoma evacuation.

As a result of rehabilitation in another hospital, he was discharged from the hospital on his own, leaving behind mild limb numbness.

## Discussion

In this study, we evaluated the clinical outcomes of CMEL performed in 33 consecutive cases in a single center and by a single surgeon. Some patients simultaneously underwent CMEL combined with foraminal decompression when radiculopathy coexisted. CMEL was applied for continuous or skip levels if necessary. Although the follow-up period in this study was relatively short (less than one year), clinical results seemed satisfactory. Progressive postoperative kyphosis was not observed, and cervical alignment was well maintained. Although postoperative epidural hematoma occurred only in one case, the other patients were discharged from our clinic the following day without any complications, including severe neck pain after surgery.

Minamide et al. initially proposed CMEL in 2010, and this method has been reported with excellent postoperative clinical and radiological outcomes [10]. However, to our knowledge, only a limited number of papers regarding CMEL have been described [10-12]. In the first report of CMEL, Minamide et al. applied this operation for patients with cervical myelopathy at a single or more cervical levels [10]. Those patients with tumors, trauma, severe ossification of the posterior longitudinal ligament (OPLL), rheumatoid arthritis, pyogenic spondylitis, destructive spondylo-arthropathies, and other combined spinal lesions were all excluded [10,11]. Minamide et al. described that alternative surgical fashion, such as conventional cervical laminoplasty or anterior corpectomy, could be appropriate for patients with OPLL or developmental canal stenosis [10]. Although patients with continuous OPLL or those requiring a posterior shift of the spinal cord were excluded, three patients with segmental-type OPLL were successfully treated in our study. CMEL may be a useful option in managing segmental-type OPLL.

Progressive kyphotic change after posterior cervical decompression can induce secondary problems [6]. In our study, however, the chronological change of the median C2-C7 angle was 6.2° to 8.1°. This result suggests cervical alignment following CMEL can be maintained or relatively lordotic. Previously, we also reported a case treated with CMEL in which postoperative decreased kyphosis was observed. Although the patient was followed only for nine months after surgery, the C2-C7 angle changed from -13° to -5° [15]. To avoid progressive kyphotic change, the nuchal ligament should be preserved. We drilled and removed the laminae and spinous processes, depending on the compression location of the spinal cord, but we did not damage the continuity of the nuchal ligament. This surgical fashion seems favorable to prevent postoperative kyphotic change. Minamide et al. described that the C2-C7 angle changed from 13.1 ± 14.0° to 13.5 ± 14.9° in 51 patients followed for six to 56 months after CMEL [10]. Dahdaleh et al. measured the Cobb angle at the level treated with CMEL in 10 patients. The Cobb angle changed from -0.43 ± 1.9° to 0.25 ± 1.6° at the average follow-up of 18.9 ±32.1 months [16]. Minamide et al. evaluated the C2-C7 angle of 71 patients followed for two years after CMEL. The preoperative C2-C7 angle was maintained from 12.3 ± 10.7° to 13.6 ± 10.6° [11]. Minamide et al. also reported that lordotic maintenance and change were observed five years after CMEL [12].

Regarding postoperative complications of CMEL, the following events have been reported, such as dura tear, epidural hematoma, and C5 nerve root palsy [10-12]. We experienced only an epidural hematoma in a single case. Before this case, we routinely removed the drainage tube the day after surgery, regardless of the amount of drained serum, if any remarkable findings were not observed on the MRI. However, after this case of postoperative epidural hematoma, we altered the protocol of removing the drainage tube; it was removed when the amount of the daily drained serum decreased below 30 ml. The following day after surgery, patients were discharged from our clinic with the drain inserted, and the drainage tube was removed two or three days later. No postoperative epidural hematoma occurred, and no infectious complications were observed either.

As for posterior cervical decompression surgery, laminoplasty and laminectomy are established methods. However, in daily practice, some patients do not wish for those typical operations because of psychological stress and surgical invasiveness. Especially when the myelopathic symptoms remain insignificant, patients can feel reluctant to agree with surgical treatment until progressive myelopathy results in gait disorders and clumsiness. As a result, persistent symptoms lasting even after surgical treatment may lead to postoperative dissatisfaction among patients. The less invasive nature of CMEL may reduce the patients’ psychological stress and lead to surgical intervention in the early period and good outcomes.

CMEL can be an effective and safe method to resolve cervical myelopathy and radiculopathy. The reports regarding CMEL are still limited, although this surgery seems to be widely diffused in daily practice. This is probably because of the specific technique of CMEL requiring surgeons’ training and experience. However, CMEL can be a useful and safe method with appropriate postoperative management of patients, even in a clinic.

Limitations

There are some limitations in this study. First, only a limited number of patients postoperatively followed in a short period were enrolled in this study. Therefore, further study, including a large number of patients, should be warranted. A long-term outcome should also be evaluated. Besides, we did not include those patients with perioperative high risks or significant motor weakness requiring rehabilitation. Thus, our results should not be overestimated. Second, we used MacNab scores to assess postoperative outcomes. We used this simple assessment because of the insufficient number of staff working in our clinic. A widespread qualitative evaluation, such as the Japan Orthopaedic Association cervical myelopathy evaluation questionnaire, should be used as an objective evaluation. In this regard, subjective bias was not completely excluded. Thirdly, in this study, we did not evaluate the cost-effectiveness of CMEL practiced in a private clinic. Lastly, we did not compare the outcomes of patients treated with CMEL in this study with those treated with typical cervical laminoplasty or laminectomy. This topic should be approached in further research.

## Conclusions

In this study, we reported our clinical outcomes of CMEL performed in 33 consecutive patients with spondylotic myelopathy, radiculopathy, and OPLL. CMEL can be safely and effectively performed for patients with those pathologies even in a private clinic when patients do not have the perioperative high risks. Our study seems to add clinical significance to CMEL because of the insufficient number of papers concerning CMEL. 
